# Fungal Community Assembly in Standing Deadwood: Stochastic vs. Deterministic Processes Across Decay Stages*

**DOI:** 10.1111/1758-2229.70208

**Published:** 2025-10-22

**Authors:** Bo Chen, Xing‐Ping Liu, Hua Lu, Feng‐Gang Luan, Zi‐Liang Zhang, Jiang‐Tao Zhang

**Affiliations:** ^1^ Provincial Key Laboratory of Conservation Biology, School of Forestry Jiangxi Agricultural University Nanchang Jiangxi China

**Keywords:** chemical properties, deadwood mycobiota, decay classes, deterministic processes, masson pine, stochastic processes

## Abstract

A growing body of evidence suggests that fungi play an essential role in the decomposition process of deadwood. However, the dynamic pattern of fungal community assembly in deadwood remains poorly understood. Here, we employed a ‘space‐for‐time’ substitution approach in the local forest to track shifts in the deadwood mycobiota during decay. The results indicated that fungal community diversity increased from decay classes I to III, then decreased from decay classes III to IV. A high degree of structural similarity in fungal communities could occur between decay classes I and II, or decay classes III and IV. Fungal community assembly in decay classes III and IV was more governed by stochastic processes than in decay classes I and II. Moreover, we identified the six most important biomarkers and established a model that associates these biomarkers with decay classes using a random forest analysis. The chemical properties of deadwood substrates were determined to be the important driver of fungal community assembly and diversity. Our work provides novel insights into changes and the generation of fungal communities within deadwood.

## Graphical abstract

*Pinus massoniana* , a key conifer in southern China, often succumbs to pests and strong winds, leaving abundant standing deadwood. However, under local field conditions, the mechanism of fungal community assembly within this deadwood remains poorly investigated. This study reveals the dynamics and drivers of these communities within *Pinus massoniana* deadwood during decomposition.

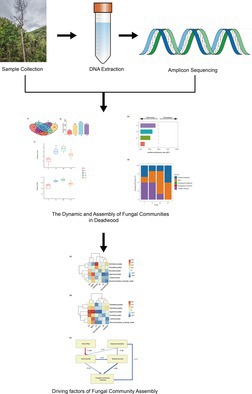

## 1 Introduction

The world's deadwood is an important carbon reservoir, accounting for approximately 8% of the global forest carbon stock (Fernández‐Martínez et al. 2014; Seibold et al. 2021). The dynamics of its carbon stock and emissions hinge on the progression of decay. Fresh wood primarily comprises lignin and cellulose, with lignin as a strong barrier to polysaccharide degradation (Pastorelli et al. 2017; Seibold et al. 2021). The degradation of lignified polysaccharides relies on highly specialised enzymatic and non‐enzymatic processes, predominantly found in certain microbial groups. Among these, wood‐decay fungi are the primary decomposers of lignin and cellulose in decaying wood, driven by their secretion and activity of oxidative and hydrolytic enzyme systems (Kirk and Farrell 1987; Floudas et al. 2012; Riley et al. 2014; Morgenstern et al. 2014). The decay of deadwood by fungal communities is driven by their assembly process, which operates through a combination of stochastic and deterministic processes (Dini‐Andreote et al. 2015; Gora and Lucas 2019; Skelton et al. 2019; Tláskal et al. 2021; Shi et al. 2024a, 2024b). However, few studies have directly focused on the contribution of deterministic and stochastic processes to fungal community assembly in deadwood.

The relative importance of deterministic and stochastic processes in fungal community assembly shifts dynamically throughout deadwood decomposition. Deterministic processes, including competition, environmental filtering, ecological selection, species‐sorting, and niche effects, play a more important role in early decomposition stages due to strong selective pressures from substrate traits, such as impermeability, high lignin, and low nitrogen in fresh wood (de Boer et al. 2005; Daniel et al. 2019). In contrast, stochastic processes might become increasingly dominant during later decomposition stages as substrate modification by microbes (Baldrian 2017). However, this general pattern varies with tree species and their distinct wood traits. A case in point is Masson pine ( *Pinus massoniana* Lamb), one of the most important native conifer trees in southern China, which adapts well to acidic, infertile soils. The decomposition of *Pinus massoniana* is characterised by the pronounced accumulation of lignin. This persistent lignin enrichment may impose strong selective pressure on the deadwood microbiota due to the limitation of readily available resources (Shi et al. 2024a, 2024b). Consequently, we hypothesize that deterministic processes will increasingly influence deadwood mycobiota as *Pinus massoniana* decomposition proceeds.

Changes in deadwood mycobiota composition have been widely explored within deterministic processes, such as the driving factors of wood traits. These studies consistently reveal that Basidiomycota and Ascomycota maintain dominance as the two most abundant fungal phyla throughout decomposition (Shi et al. 2024a, 2024b; Chen et al. 2025). Ascomycota can utilise readily available nutrients (carbon compounds, hemicellulose, and cellulose), while Basidiomycota has established competitive advantages by utilising a broader range of wood fibre, particularly lignin, which is more difficult to use (Boddy and Watkinson 1995). However, several studies have shown that Ascomycota has significantly higher species richness and relative abundance than Basidiomycota in deadwood (Huang et al. 2022; Shi et al. 2024a, 2024b). Therefore, these findings compel a reconsideration of the precise influence of wood fibre on the competitive balance between Ascomycota and Basidiomycota.

Despite increasing research efforts, the influence of deadwood properties on microbial community assembly remains poorly understood. To the best of our knowledge, there has been no report about microbial community assembly during the decay of standing masson pine wood (Shi et al. 2024a, 2024b). In this study, taking advantage of the essential gap in the literature, we focused on masson pines with standing deadwood. We performed a space‐for‐time field trait analysis to track the deadwood mycobiota shifts during decay progression. The association between deadwood mycobiota and decay classes was assessed using the Random Forests model. We used these data to determine whether deterministic processes increasingly influence deadwood mycobiota as *Pinus massoniana* decomposition proceeds by using the null, neutral model. Furthermore, the relationships between fungal taxa and wood traits were explored using correlation analysis. Lastly, the relative contribution of these wood traits to fungal community diversity is determined using a structural equation model.

## 2 Materials and Methods

### 2.1 Study Sites

The study was conducted at Huangyun Reservoir, Jiangxi Province, China (24.633° N, 114.353° E; altitude: 400 m), characterised by a subtropical monsoon climate (mean annual temperature: 18°C; precipitation: 1700 mm) and subtropical red soil. The forest is dominated by Masson pine ( *Pinus massoniana* ), covering an area of about 2 ha, which has been severely impacted by pine wood nematode (*Bursaphelenchus xylophilus*), leading to extensive deadwood production. Pinewood nematode‐infested trees were identified and removed in local forest management to control disease spread. However, there are still some uncleaned dead trees in the form of standing dead trees.

### 2.2 Sample Collection

Standing deadwood was categorised into five decay classes according to the classification system described by Yan et al. (2005). Due to the difficulty in determining whether deadwood in decay class V originated from standing deadwood (see Table S1), we selected standing deadwood from the first four decay classes (excluding decay class V) with a diameter at breast height (DBH) of approximately 15 cm and similar site conditions. From the base of 16 deadwood samples (four replicates per decay class), wood discs with a thickness of 15 cm were cut at a height of 30 cm above the ground. After removing the bark, the wood discs were further cut into 4 cm pieces, fully mixed, and stored individually in sterile bags. These bags were immediately placed in incubators at approximately 4°C. In the laboratory, a portion of the wood pieces was cryogenically milled using a mortar and stored at −80°C for subsequent amplicon sequencing. Another portion of the wood pieces was ground into powder using a grinding machine for chemical analysis.

### 2.3 DNA Extraction and High‐Throughput Sequencing

Total genomic DNA was extracted from 16 deadwood samples using the CTAB method. DNA quality and size were assessed by electrophoresis on 1% agarose gels, and concentrations were standardised to 1 ng/μL with sterile water. The fungal ITS region was amplified with primers ITS1‐1F‐F (5′‐CTTGGTCATTTAGAGGAAGTAA‐3′) and ITS1‐1F‐R (5′‐GCTGCGTTCTTCATCGATGC‐3′), targeting hypervariable regions. PCR products were labelled with unique error‐correction barcodes for sample identification. Successful amplification was verified by running PCR products on 2% agarose gels with 1× TAE buffer. After purification, amplicons were quantified, pooled in equimolar ratios, and subjected to high‐throughput sequencing on the Illumina NovaSeq 6000 platform. The resulting raw FASTQ files were used for subsequent microbial community profiling.

### 2.4 Deadwood Chemical Properties Analysis

The fibre composition of deadwood was determined with a crude fibre analyser (F6800, Alva, China). Initially, cellulose, hemicellulose, and lignin were retained by treating the samples with a Neutral Detergent Fibre (NDF) solution, which removes soluble sugars, proteins, and lipids. Subsequently, hemicellulose was removed by further treating the NDF residue with an Acid Detergent Fibre (ADF) solution, leaving behind cellulose and lignin. Finally, lignin was isolated by treating the ADF residue with concentrated sulfuric acid, which dissolves cellulose. The contents of cellulose, hemicellulose, and lignin were calculated based on the mass differences at each step (Van Soest et al. 1991). Total nitrogen (TN) content of deadwood samples was determined with a nitrogen analyser (KN‐520, Alva, China). Total phosphorus (TP) content was quantified on a full‐wavelength microplate reader (SpectraMax 190, Molecular Devices, America). Sample digestion for total carbon determination was performed using an oil bath (HH‐S, LangYue Instrument, China) (Wu et al. 2014).

### 2.5 Bioinformatics Analysis

Raw sequencing data were rigorously processed to ensure ASV (Amplicon Sequence Variant) accuracy. FASTQ files were analysed in QIIME2 (version 2021.2), where sequences underwent demultiplexing, quality filtering, trimming, denoising, and merging. Chimeric sequences were detected and removed via the QIIME2‐integrated VSEARCH tool (version 2.7.0), followed by ASV clustering at 97% similarity. Taxonomic annotation was performed using USEARCH (version 11.0.667) against the RDP database. Contaminating mitochondrial and chloroplast sequences were eliminated through text‐based filtering in Linux to generate final taxonomy tables.

Alpha diversity analysis was calculated using R software (version 3.6.2). The diversity index was compared among decay levels using the least significant difference (LSD) test within the ‘vegan’ R package (version 2.5.7). In order to assess fungal community dissimilarities among different decay levels, Bray‐Curtis distances based on ASV abundance profiles were computed using ‘vegan’ R package (version 2.5.7) and visualised through Principal Coordinates Analysis (PCoA) through ‘ggplot2’ R package (version 2.2.1). Stacked bar plots were visualised using the ‘ggplot2’ R package (version 3.3.6). Enriched species across groups were identified by Wilcoxon rank‐sum tests (applied to ASVs with median relative abundance > 0.001%), with FDR‐adjusted significance threshold (*p* < 0.05) (Li et al. 2023). To acquire the best discriminant performance of taxa across decay classes in the local forest, the relative abundance of fungal classes was regressed against decay classes with default parameters using the ‘randomForest’ R package (version 4.6.14) in R software (version 4.0.0). Ten‐fold cross‐validation was set to evaluate the importance of fungal biomarkers across decay progression (Zhang et al. 2018; Li et al. 2023). Here, the Random Forest machine‐learning method was first employed to assess decay progression.

The Modified Stochasticity Ratio (MST), a metric derived from a null model‐based statistical framework, aimed to assess the relative importance of deterministic and stochastic processes in deadwood mycobiota (Guo et al. 2021; Liu et al. 2021; Cheng et al. 2024). The 50% threshold for the MST index was defined as a distinction between deterministic dominance (MST < 50%) and stochastic dominance (MST > 50%) community assembly. The MST analysis based on Bray‐Curtis distances was performed using the ‘NST’ R package (version 3.1.10). Moreover, the β nearest‐taxon index (βNTI) and modified Raup‐Crick index (RC_Bray_) were applied to classify ecological assembly processes in deadwood mycobiota, such as dispersal limitation, drift, homogeneous dispersal, homogeneous selection, and variable selection. Specifically, detected taxa were first assigned to various groups (called ‘bin’) to quantify ecological processes. For each bin, a value of βNTI > +2 indicates that community assembly is mainly governed by variable selection, while βNTI < −2 represents homogeneous selection. The |βNTI| < 2 demonstrates that stochastic processes primarily control community assembly. Additionally, RC_Bray_ values were used to facilitate segmenting the stochastic processes. The contribution of dispersal limitation is determined by |βNTI| < 2 and RC_Bray_ > +0.95, whereas RC_Bray_ < −0.95 represents homogeneous dispersal. The |βNTI| < 2 and |RC_Bray_| < 0.95 indicate undominated processes, including weak selection, weak dispersal, diversification, and drift. Using the ‘microeco’ R packages (version 1.13.0), the βNTI and RC_Bray_ based on null model tests were carried out (Zhou and Ning 2017; Zhang et al. 2019). Notably, current statistical methods for evaluating stochastic and deterministic processes cannot yet provide fully quantitative results, and sometimes even produce contradictory outcomes. Nevertheless, they are all capable of revealing the changing trends in microbial community assembly processes (Wang et al. 2025).

Spearman and Pearson's correlations between taxa and deadwood chemical properties were calculated using the ‘psych’ R package (version 2.4.12). Correlation heatmaps were formed using the ‘pheatmap’ R package (version 1.0.12) (Yang et al. 2023). Partial least squares path model (PLS‐PM) analysis was used to assess further direct and indirect effects of deadwood chemical properties on fungal communities, and run with ‘plspm’ package (version 0.5.1). The final model passed goodness‐of‐fit (GOF) metrics requirements, following the baseline model that underwent continuous modification and optimization (Wen et al. 2022).

## 3 Results

### 3.1 Fungal Diversity and Community Structure Among Different Decay Classes

**FIGURE 1:**
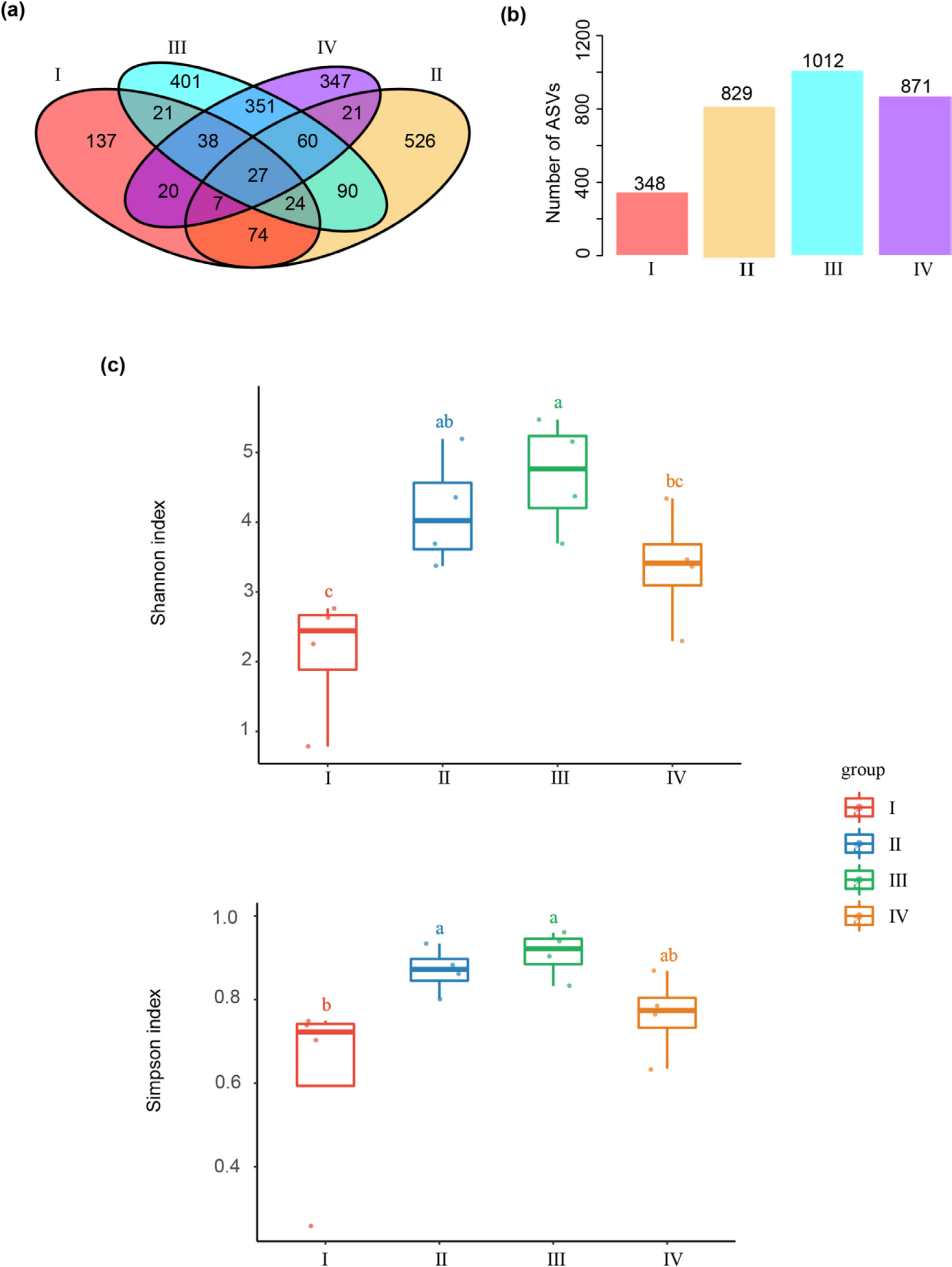
Comparison of deadwood microbial community structures during decay progression. (a, b) Amplicon Sequence Variant (ASV) Venn diagram of deadwood fungal community describing the species‐level difference among four decay classes. (c) Alpha diversity indexes of fungal community shift with different decay classes.

A total of 2144 ASVs were identified from 16 samples of *Pinus massoniana* deadwood across four decay classes (I to IV). The Venn diagram shows that 27 core ASVs, represented in all decay class levels, mainly belonged to phylum Ascomycota. There were 132 ASVs overlapped between decay class I and II, accounting for 37.93% in decay class I (*n* = 348) and 15.92% in decay class II (*n* = 829), respectively. Furthermore, 201 common ASVs were found between decay classes II (*n* = 829) and III (*n* = 1012), representing 24.25% and 19.86% of each decay class. Additionally, 476 common ASVs were detected from class III (*n* = 1012) and IV (*n* = 871), accounting for 47.04% and 54.65%, respectively (Figure 1a,b). Alpha diversity analysis, consisting of Shannon diversity, Simpson diversity, Pielou evenness, ACE, Chao1, and observed_species, revealed two key trends of the deadwood mycobiota diversity: a significant increase from decay class I to II, and no significant difference between decay class II and III (Figure 1c).

**FIGURE 2:**
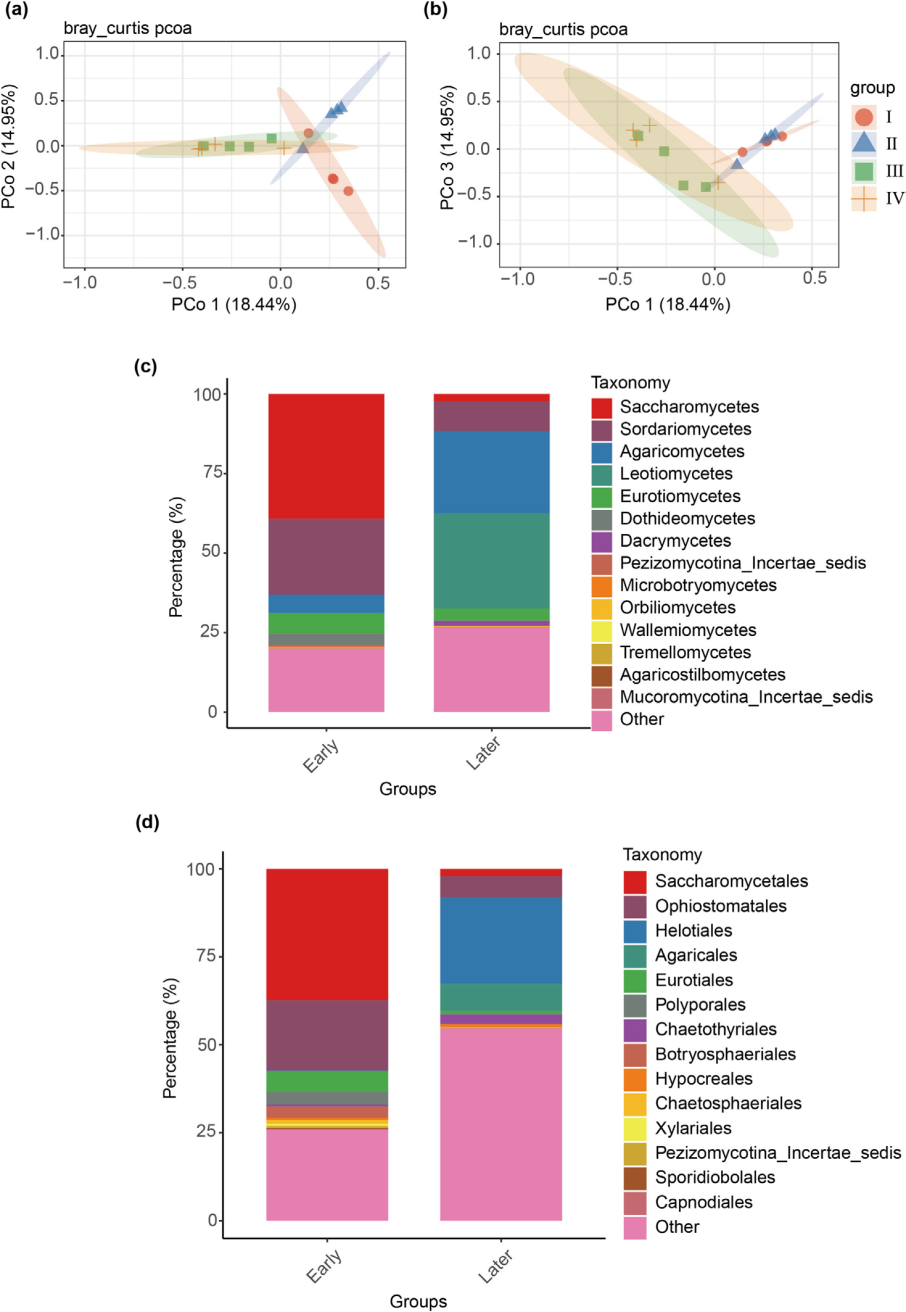
Comparison of deadwood microbial community structures during decay progression. (a, b) Principal coordinate analysis (PCoA) of deadwood fungal communities across different decay classes in local forests. (c) The class‐level fungal distribution. Each bar represents eight biological replicates. (d) Order‐level fungal distribution. Each bar contains eight biological replicates.

Beta diversity analysis based on Bray–Curtis dissimilarity separated the decay classes of deadwood samples into two clusters: classes I‐II and classes III‐IV (Figure 2a,b). The confidence ellipses of class I and class II partially overlapped, whereas the ellipses of class III and class IV showed a large overlap, indicating a high degree of similarity in the later stage (Figure 2a,b). PERMANOVA confirmed a significant difference between classes II and III (*p* = 0.017, *R* ^2^ = 0.2721), but no significant difference was detected between class I and II (*p* = 0.053, *R* ^2^ = 0.2588). Furthermore, a significant difference was also detected between the classes I‐II and classes III‐IV (*p* = 0.001, *R* ^2^ = 0.1647). Given this robust statistical evidence, we defined classes I‐II as the early decay stages and classes III‐IV as the later decay stages, respectively. To further identify differences within the deadwood fungal community of the two groups, we compared the fungal community composition between these groups. Saccharomycetes and Sordariomycetes were more common in the early decay stage (39.25% and 23.83%, respectively). In contrast, Agaricomycetes (25.68%) and Leotiomycetes (30.06%) were dominant classes in the later decay stage (Figure 2c). At the order level, Saccharomycetales and Ophiostomatales were predominant in the early decay stage, representing 37.37% and 19.95% of the community, respectively. Meanwhile, the proportion of Helotiales was 24.63% in the later decay stage (Figure 2d). Moreover, differential abundance analysis showed that 62 ASVs (accounting for 9.34% of total sequencing reads), representing five classes (Eurotiomycetes, Leotiomycetes, Sordariomycetes, Agaricomycetes, Mucoromycotina_Incertae_sedis), were significantly enriched in the later decay stage as compared to the early decay stage (Figure S1). Additionally, four species (*Sporothrix lignivora*, *Penicillium meleagrinum*, *Mortierella chlamydospore*, and *Hypocrea lixii*), enriched in the later decay stage, were identified.

### 3.2 Fungal Community Assembly Mechanisms Among Different Decay Classes

**FIGURE 3:**
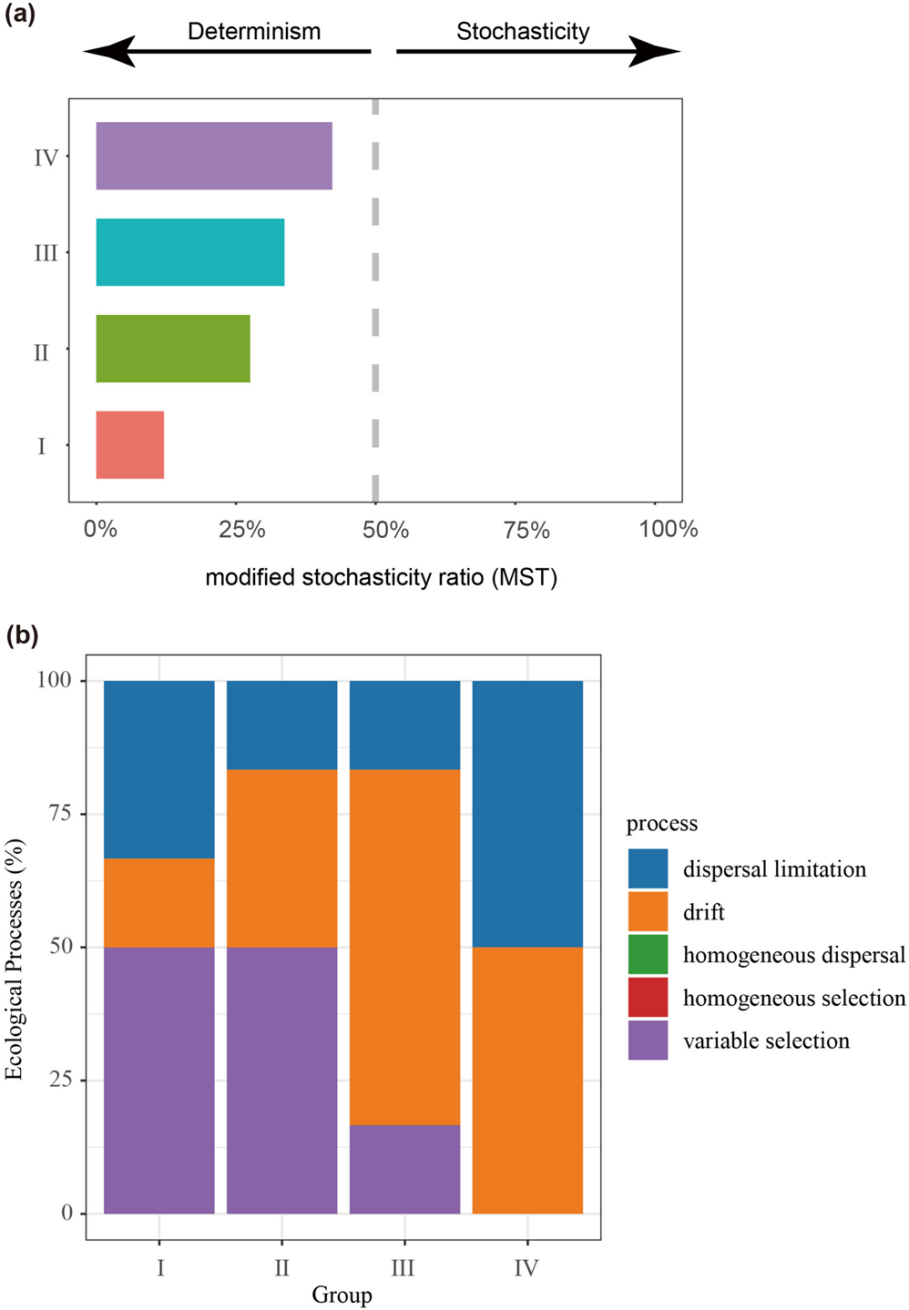
The influence of stochastic and deterministic processes on fungal community assembly during decay progression. (a) The modified stochasticity ratio (MST) of fungal communities under different deadwood decay classes based on Jaccard dissimilarity with 50% as the boundary point between more deterministic (< 50%) and more stochastic (> 50%) community assembly. (b) The proportion of different ecological processes within different decay classes using null model tests based on the βNTI and RCbray.

The MST ratio, calculated using Jaccard dissimilarity across four decay classes, revealed that the fungal community of all the decay classes was greatly governed by deterministic processes (MST < 50.00%), in which decay class IV showed the lowest deterministic processes (Figure 3a). Moreover, the null model based on βNTI and RC_bray_ revealed that variable selection was the dominant ecological process in decay classes I and II. In contrast, drift dominates the deadwood fungal community assembly in decay classes III and IV (Figure 3b). The relative importance of fungal assembly processes indicated that variable selection (50.00%), dispersal limitation (33.33%), and drift (16.67%) contributed to the microbial assembly processes in decay class I. From decay classes I to IV, the relative contribution of variable selection declined to a negligible level, while both drift and dispersal limitation rose to 50.00% (Figure 3b).

### 3.3 The Influence of Chemical Properties Within Deadwood on Fungal Assembly Mechanisms and Fungal Diversity

**FIGURE 4:**
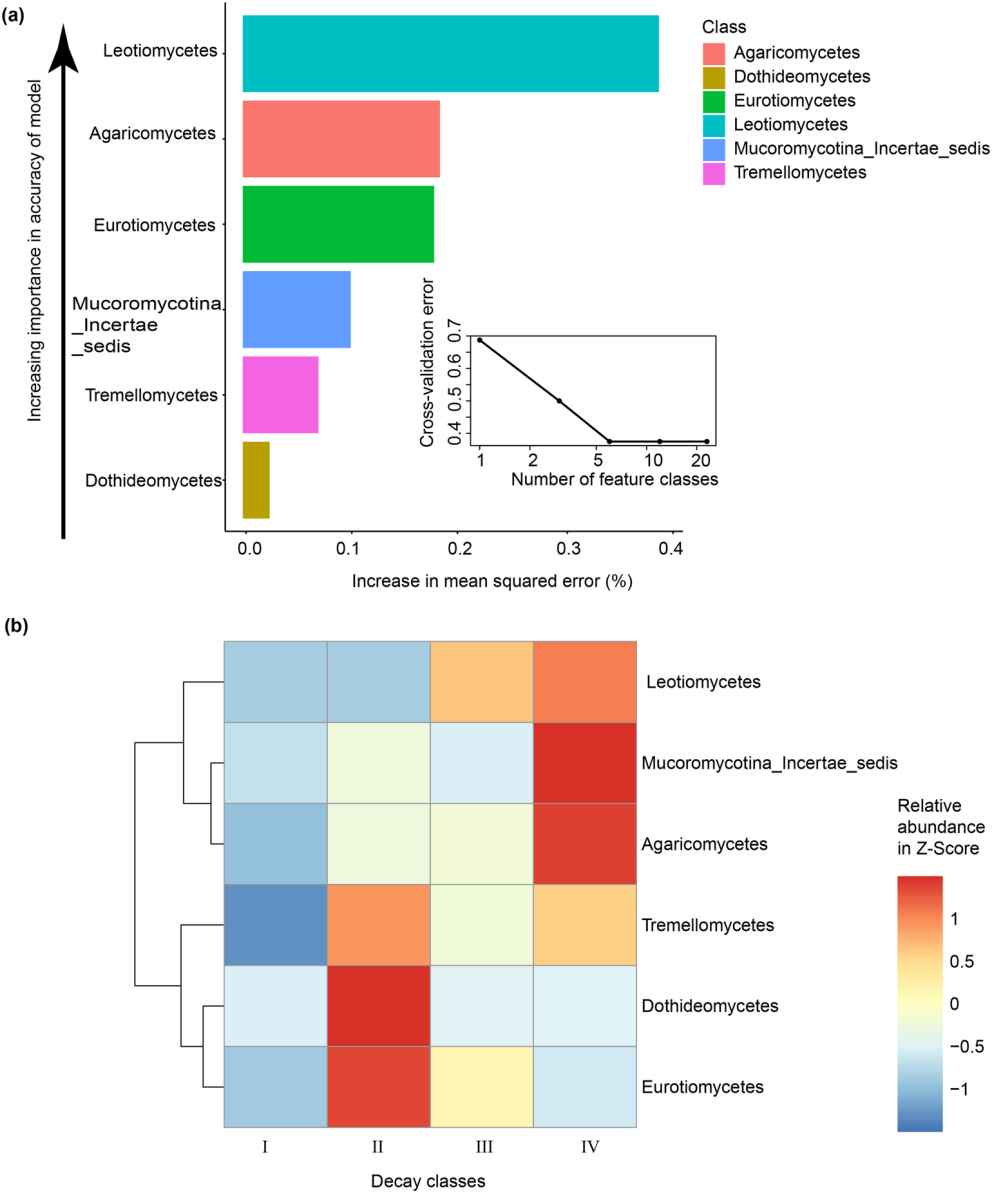
Fungal taxonomic biomarkers across decay classes. (a) Top 6 important fungal classes identified with applying Random Forest Regression. These biomarkers were ranked by descending order of importance for model accuracy. (b) Heatmap shows changes in the relative abundance of top 6 important fungal classes across decay classes in the local forest.

Functional fungal communities dominate deadwood decay and are modified by wood substrate traits (Huang et al. 2022; Shi et al. 2024a, 2024b). To identify the important fungal classes as biomarker taxa associated with decay stages, the relative abundance of deadwood fungi at the class level was regressed to construct a model evaluating the importance of fungal classes. The model explained 60.23% of the variation in deadwood mycobiota following decay classes in the local forest. The cross‐validation error rate was minimal, and the highest stability was observed when the top 6 important taxa were used (Figure 4a). Thus, we defined the top 6 important taxa at the class level as putative biomarkers in this model. The list of the top 6 important taxa at the class level, in order of increasing importance in the accuracy of this model, is shown in Figure 4a. Additionally, the Leotiomycetes, Mucoromycotina_Incertae_sedis, and Agaricomycetes remained at high levels in decay class IV, while Tremellomycetes, Dothideomycetes, and Eurotiomycetes presented high levels in decay class II (Figure 4b).

**FIGURE 5:**
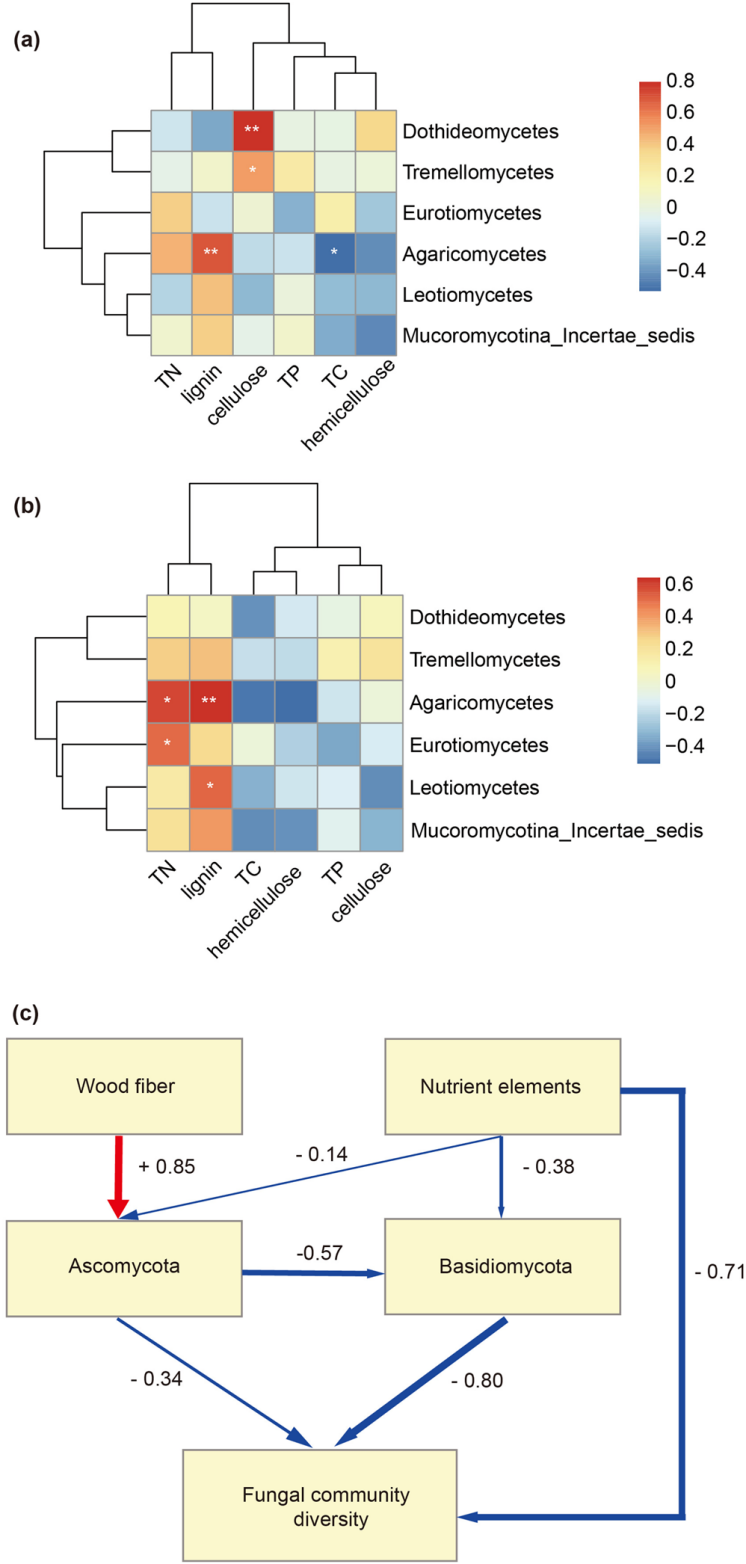
(a, b) Correlation analysis between chemical properties and class‐level biomarkers based on Pearson and Spearman correlation coefficients, respectively. The legend shows a series of colour gradients and notes the corresponding numerical range, with blue representing negative correlations and red showing positive correlations. Significance levels are denoted by asterisks: *p < 0.05, **p < 0.01. TC means total carbon, TN means total nitrogen, and TP means total phosphorus. (c) Partial Least Squares Path Modelling (PLSPM) describing the effect among wood fibre, nutrient elements, fungal community diversity, and biomarkers of Ascomycota and Basidiomycota. Red and Blue arrows show positive and negative effects, respectively. The total effect values corresponding to each driver are indicated near the lines.

To further assess the contribution of variable selection during decay progression, we examined the response of fungal communities to the chemical properties of deadwood. Specifically, the average content of lignin increased gradually during decay progression, while the average content of total cellulose peaked in decay class II (Figure S2). Moreover, the average content of total carbon (TC) decreased gradually from decay classes II to IV, whereas the average content of total nitrogen peaked in decay class II (Figure S3). In Pearson correlation analysis, Agaricomycetes showed a positive correlation with lignin but a negative correlation with TC. Dothideomycetes and Tremellomycetes were positively correlated with cellulose (Figure 5a). In Spearman correlation analysis, Agaricomycetes and Leotiomycetes were positively correlated with lignin. Agaricomycetes and Eurotiomycetes were positively correlated with TN (Figure 5b).

To investigate potential relationships between fungal biomarkers and fungal diversity in deadwood, Pearson and Spearman correlation analyses were conducted according to the relative abundance of fungal biomarkers and the alpha diversity index. In Pearson correlation analysis, Agaricomycetes was negatively correlated with Shannon, Simpson, and Pielou evenness (Figure S4a). In Spearman correlation analysis, all biomarkers except Agaricomycetes were positively correlated with all alpha indices (Figure S4b). The comprehensive relationships between biomarkers (Ascomycota and Basidiomycota), fungal diversity, and deadwood chemical properties (wood fibres and common nutrient elements) were profiled by PLSPM analyses. The model best fit was achieved with a GOF of 0.58. Wood fibres influenced the relative abundance of Ascomycota biomarkers directly (path coefficient of A = 0.85) or indirectly by their effect on fungal diversity. Additionally, nutrient elements (−0.71) exerted a strong impact on fungal diversity (Figure 5c).

## 4 Discussion

### 4.1 The Fungal Community Dynamics During Decay Progression

Fungal community in deadwood can maintain a highly similar structure across several decay classes. Previous work has shown that the fungal community structure within pine logs is significantly similar across several decay stages; however, these studies have focused on lying deadwood (Pastorelli et al. 2017; Shi et al. 2024a, 2024b). We collected standing deadwood samples spanning distinct decay stages (I–IV) from a single forest site to minimise environmental heterogeneity. In our data, fungal communities showed no significant difference between decay classes I and II or between classes III and IV. In contrast, a statistically significant difference was detected between classes II and III, suggesting a critical transition in deadwood mycobiota between decay classes II and III. Moreover, the groups of the decay classes I–II and decay classes III–IV were significantly different from each other (Figure 2a,b). Therefore, we classified deadwood decomposition into early (classes I and II) and later (classes III and IV) decay stages based on this change.

Core ASVs have been reported to dominate the microbial community across decay classes (Gupta et al. 2022; Shi et al. 2024a, 2024b). However, a limited number of core ASVs were observed in our findings, primarily affiliated with the phylum Ascomycota (Figure 1a). Agaricomycetes and Leotiomycetes were the dominant classes in the later decay stage, while Saccharomycetes and Sordariomycetes were more common in the early decay stage. Our analysis identified enrichment of several fungal species within the later decay stage, such as *Sporothrix lignivora*, *Penicillium meleagrinum*, and *Hypocrea lixii*. These species were generally divided into groups depending on their strong decay abilities: (i) Cellulose‐decomposing fungi, including *Sporothrix lignivora*, *Penicillium meleagrinum* (Shrestha et al. 2015; Boruah et al. 2016), and (ii) Lignin‐decomposing fungi, including *Hypocrea lixii* (He et al. 2023). A key question for future research is to identify the factors driving the succession of these functional species during decay progression. Unraveling these drivers will help us better reveal the triggering mechanism for functional fungi enrichment, thereby further providing environmentally friendly solutions for the disposal of forest waste.

### 4.2 Variations of Chemical Properties Influencing Fungal Community Assembly and Fungal Diversity

The Random Forest analysis identified that the relative abundance of Agaricomycetes was highest in decay class IV (Figure 4b). We further examined the chemical properties within deadwood associated with decay classes. Consistent with previous studies (Shi et al. 2024a, 2024b; Stokland and Alfredsen 2024), we found that the average lignin content increased gradually as the decay classes increased, with content significantly higher in decay class IV than in decay classes I and II (Figure S2a). Furthermore, correlation analysis indicated that the abundance of Agaricomycetes was significantly positively correlated with lignin (Figure 5a,b). Thus, the role of Agaricomycetes as decomposers might be more critical in decaying deadwood within decay class IV by degrading the accumulated lignin. Our PLSPM analysis showed that wood fibre had direct positive effects on Ascomycota (Dothideomycetes, Eurotiomycetes, and Leotiomycetes) (Figure 5c), which may be related to their capacity for cellulose degradation (Voříšková and Baldrian 2013; Huang et al. 2022). Moreover, both Ascomycota and Basidiomycota had direct negative effects on fungal community diversity, while Ascomycota also showed indirect effects. This negative relationship between certain taxa and overall diversity is partly supported by Yang et al. (2016), who found that the decay rate of deadwood may be inhibited by higher fungal diversity but promoted by certain decay taxa. Together, these results suggest that the chemical properties of deadwood likely conferred a competitive advantage to certain taxa during decomposition, with a subsequent limitation on fungal community diversity.

### 4.3 Assembly Mechanisms of Fungal Community Within Deadwood Across Different Decay Classes

A primary objective in microbial ecology is to understand the rules of microbial community assembly, which dictate biodiversity and composition, and ultimately influence ecosystem function (Sun et al. 2022). Combining the MST and null model, the fungal community assembly processes within deadwood were largely governed by deterministic processes, where decay class IV showed the lowest contribution of deterministic processes (Figure 3a). We further assessed the contribution of deterministic and stochastic processes by evaluating the βNTI and RC_Bray_ values. The variable selection was dominant in shaping fungal community assembly during decay classes I and II, while the relative contribution of stochastic processes (drift and dispersal limitation) to fungal community change was dominant across decay classes III and IV (Figure 3b). This observation revealed that the influence of deterministic processes on fungal community assembly gradually diminished as the decay level increased, suggesting that the overall function of fungal communities was increasingly uncertain. This finding does not support our hypothesis that deterministic processes increasingly influence deadwood mycobiota as *Pinus massoniana* decomposition proceeds.

One potential explanation is that progressive changes in deadwood's chemical properties during decomposition diminished the role of deterministic processes in microbial assembly. Firstly, lignin can form formidable physical barriers to polysaccharides within deadwood (Vanholme et al. 2010; Skelton et al. 2019). The progressive disruption of the lignin barrier as decay advanced increased the accessibility and diversity of resources, thereby diminishing environmental stress for fungal colonists. Secondly, despite the gradual accumulation of lignin (Figure S2), decaying wood can sustainably supply fungal communities with readily accessible nutrients in the long term (Figure S3). Overall, the disruption of the lignin barrier and the subsequent stable provision of readily accessible nutrients might gradually reduce the strength of environmental filtering for fungal communities, leading to a greater influence of stochastic assembly.

## 5 Conclusions

This research confirmed that the fungal community in deadwood can maintain a highly similar structure across several decay classes and elucidated the underlying fungal community assembly mechanisms, thereby providing novel insights into changes and the generation of fungal communities. Fungal community diversity represented a dynamic associated with decay classes, increasing from class I to III and then declining from III to IV. No significant differences were observed in the fungal community structure between decay classes I and II, or between classes III and IV. The assembly of microbial communities was more driven by stochastic processes in decay classes III and IV, and by variable selection (a deterministic process) in decay classes I and II. The top six biomarker taxa correlated with decay classes were selected with random forest analyses, such as Agaricomycete. Specifically, the relative abundance of Agaricomycetes remained at high levels in decay class IV, while Agaricomycetes was positively correlated with lignin by correlation analysis, suggesting that Agaricomycetes might play a more essential role in degrading deadwood within decay class IV. The PLSM analyses results demonstrated that wood fibre, nutrient elements, Ascomycota, and Basidiomycota were directly or indirectly related to fungal community diversity within deadwood. Notably, wood fibre had a direct positive effect on Ascomycota, and both Ascomycota and Basidiomycota were direct negative drivers of fungal community diversity. These results demonstrated that deadwood properties were determined as important driving factors of fungal community assembly and diversity. However, whether these findings can be generalised to other ecosystems or seasons remains to be elucidated.

## Author Contributions

**Bo Chen:** conceptualization (equal); data curation (equal); formal analysis (equal); methodology (lead); writing – original draft (lead); writing – review and editing (equal). **Hua Lu:** conceptualization (equal); writing – review and editing (equal). **Feng‐Gang Luan:** data curation (equal); investigation (equal); writing – review and editing (equal). **Zi‐Liang Zhang:** conceptualization (equal); data curation (equal); formal analysis (equal); investigation (equal). **Jiang‐Tao Zhang:** conceptualization (equal); data curation (equal); formal analysis (equal); investigation (equal). **Xing‐Ping Liu:** conceptualization (equal); data curation (equal); funding acquisition (lead); investigation (equal); project administration (lead); visualisation (lead); funding acquisition (lead); resource (lead); writing – review and editing (equal).

## Conflicts of Interest

The authors declare no conflicts of interest.

## Supporting information


**Figure S1:** Manhattan plot showing the ASVs enriched or depleted in the later decay stage samples versus early decay stage samples. The dashed line represents the significance threshold adjusted for the false‐discovery rate, with a *p* value of less than 0.005. Significantly enriched ASVs were indicated as filled triangles; Significantly depleted ASVs were shown as empty triangles.

**Figure S2:** (a, b) The content of lignin and cellulose shifts with decay classes.

**Figure S3:** (a–c) The content of macronutrients shifts with decay classes.

**Figure S4:** (a, b) Correlation analysis between microbial community diversity and class‐level biomarkers based on Pearson and Spearman correlation coefficients, respectively. The legend shows a series of colour gradients and notes the corresponding numerical range, with blue representing negative correlations and red showing positive correlations. Significance levels are denoted by asterisks: *p < 0.05, **p < 0.01, ***p < 0.001.

**Table S1:** Standard classification system for decomposition stages.

## Data Availability

The dataset supporting this article's conclusions is included within the article and its additional files. All raw sequencing data were uploaded to the NCBI Sequence Read Archive database (Accession Number: PRJNA1237328).
