# Supplementary figures and images for: Fungal Community Assembly in Standing Deadwood: Stochastic vs. Deterministic Processes Across Decay Stages*
Source: Environ Microbiol Rep. 2025 Oct 22;17(5):e70208. doi: 10.1111/1758-2229.70208 (PMC12541549; doi:10.1111/1758-2229.70208)

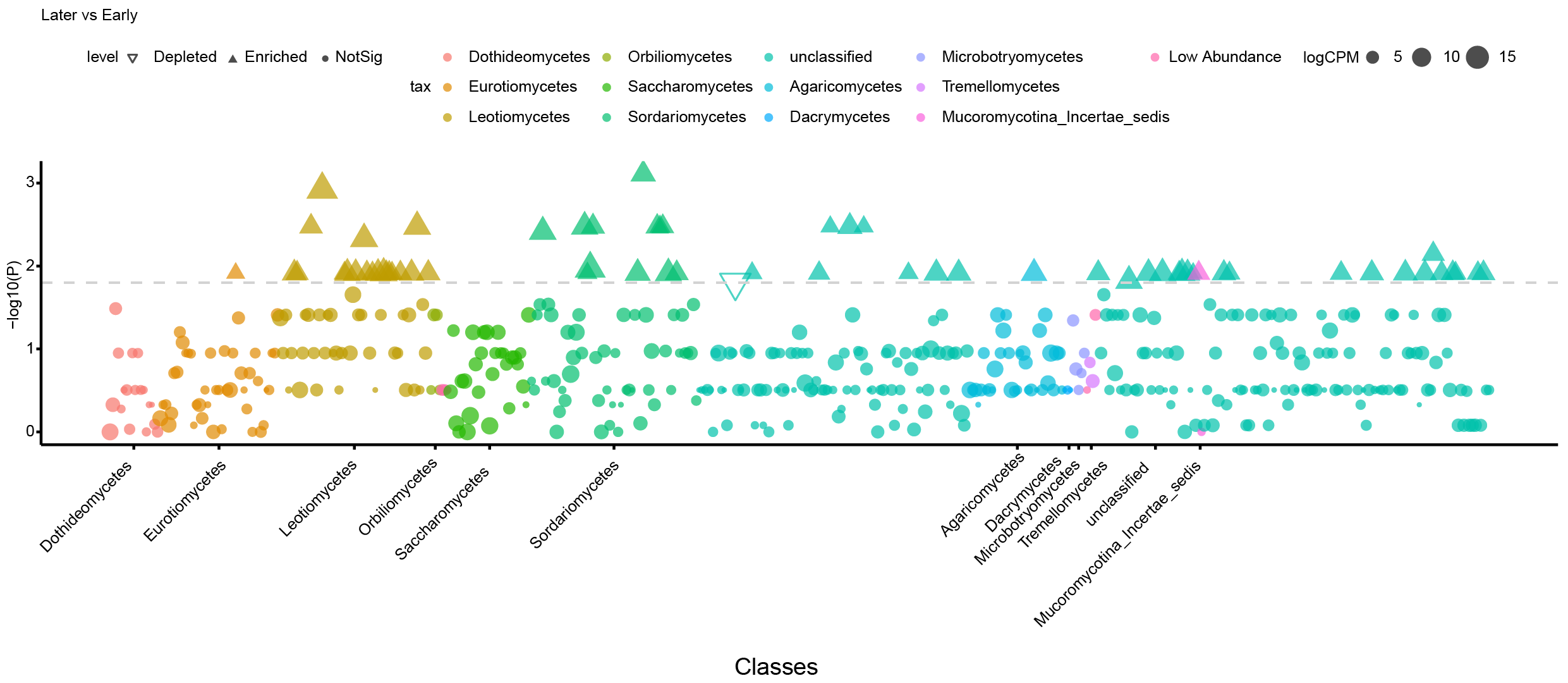

Supplement: Supplementary file 1 — Figure S1: Manhattan plot showing the ASVs enriched or depleted in the later decay stage samples versus early decay stage samples. The dashed line represents the significance threshold adjusted for the false‐discovery rate, with a p value of less than 0.005. Significantly enriched ASVs were indicated as filled triangles; Significantly depleted ASVs were shown as empty triangles. [file EMI4-17-e70208-s004.tif]

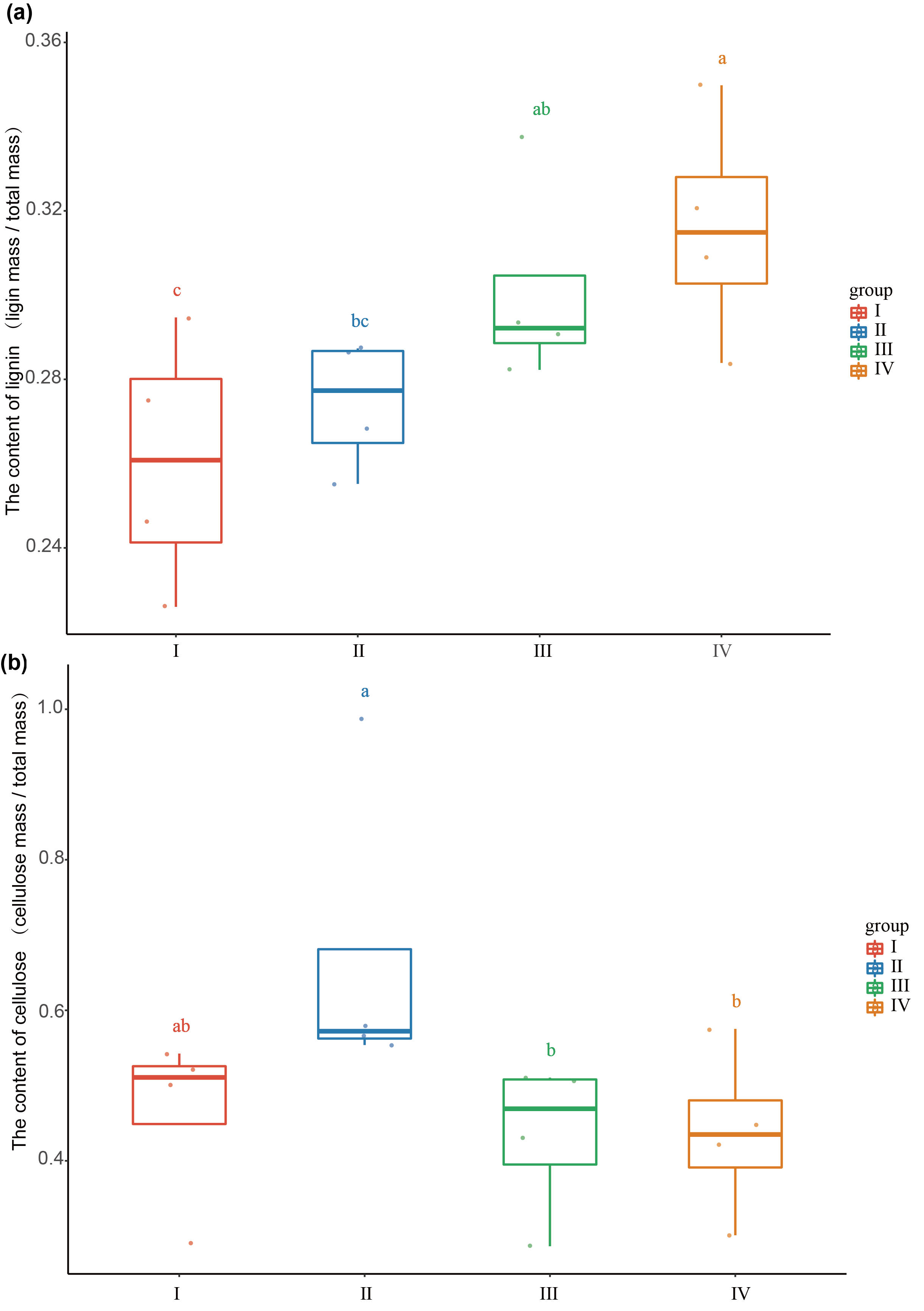

Supplement: Supplementary file 2 — Figure S2: (a, b) The content of lignin and cellulose shifts with decay classes. [file EMI4-17-e70208-s005.tif]

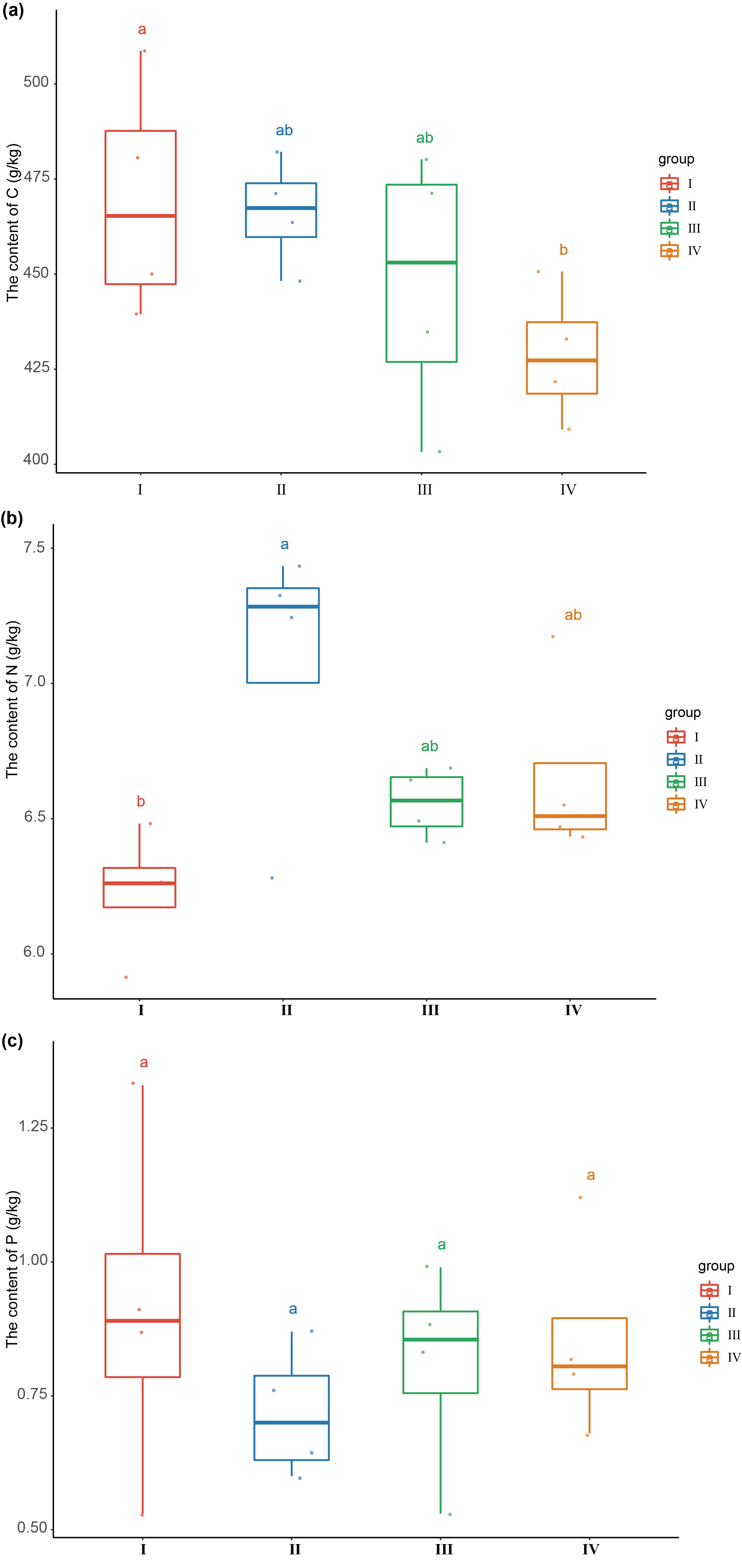

Supplement: Supplementary file 3 — Figure S3: (a–c) The content of macronutrients shifts with decay classes. [file EMI4-17-e70208-s001.tif]

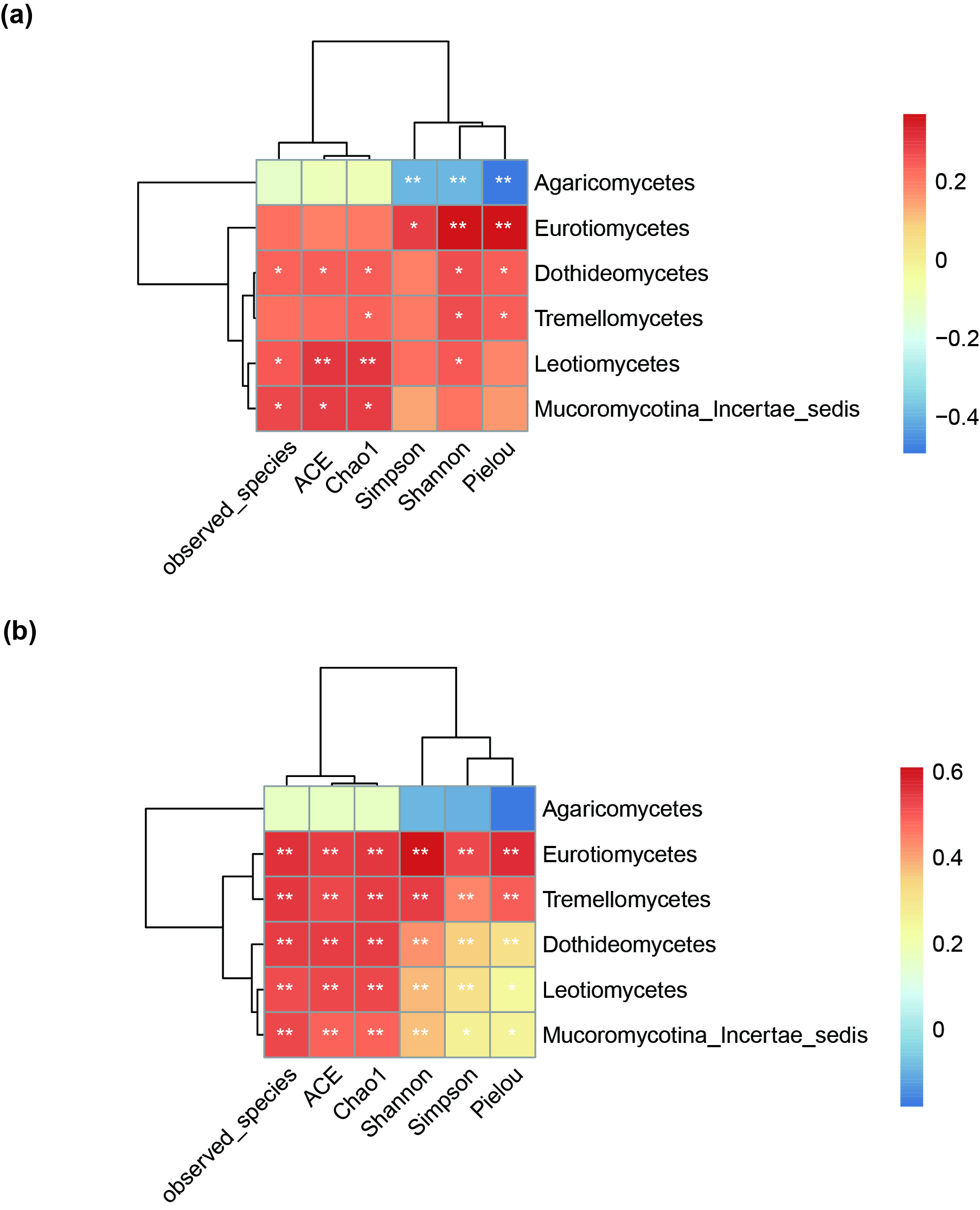

Supplement: Supplementary file 4 — Figure S4: (a, b) Correlation analysis between microbial community diversity and class‐level biomarkers based on Pearson and Spearman correlation coefficients, respectively. The legend shows a series of colour gradients and notes the corresponding numerical range, with blue representing negative correlations and red showing positive correlations. Significance levels are denoted by asterisks: *p < 0.05, **p < 0.01, ***p < 0.001. [file EMI4-17-e70208-s003.tif]
