# Supplementary material for: Fungal Community Assembly in Standing Deadwood: Stochastic vs. Deterministic Processes Across Decay Stages*
Source: Environ Microbiol Rep. 2025 Oct 22;17(5):e70208. doi: 10.1111/1758-2229.70208 (PMC12541549; doi:10.1111/1758-2229.70208)
Supplement: Supplementary file 5 — Table S1: Standard classification system for decomposition stages. [file EMI4-17-e70208-s002.docx]

Table S1 Standard Classification System for Decomposition Stages

| Type | Judgment Indicators | Decay class levels | | | | |
| --- | --- | --- | --- | --- | --- | --- |
|  |  | Ⅰ | Ⅱ | Ⅲ | Ⅳ | Ⅴ |
| Standing deadwood | Leaves | Present | Absent | Absent | Absent | Absent |
|  | Bark | Tight | Loose | Partially present | Absent | Absent |
|  | Branches & Crown | All branches present | Only large branches present | Only large branches present | Absent | Fallen wood |
|  | Trunk |  | standing, solid | standing, weak | severely weak |  |
|  | Indirect Methods | Fresh deadwood, less than 1 year | Initial decay, blade penetrates a few millimeters | Blade penetrates about 2 centimeters | Blade penetrates about 2 to 5 centimeters | Blade penetrates completely |
|  | structural integrity evaluation | Unbroken | Sapwood rotten, heartwood intact | Majority of sapwood missing, heartwood rotten | Heartwood rotten and soft | Fully soft |
